# ^1^H, ^13^C and ^15^N assignment of the paramagnetic high potential iron–sulfur protein (HiPIP) PioC from *Rhodopseudomonas palustris* TIE-1

**DOI:** 10.1007/s12104-020-09947-6

**Published:** 2020-05-15

**Authors:** Inês B. Trindade, Michele Invernici, Francesca Cantini, Ricardo O. Louro, Mario Piccioli

**Affiliations:** 1 Instituto de Tecnologia Química e Biológica António Xavier (ITQB-NOVA), Universidade Nova de Lisboa, Av. da República (EAN), 2780-157 Oeiras, Portugal; 2 Magnetic Resonance Center (CERM), Department of Chemistry and Consorzio Interuniversitario Risonanze Magnetiche Metallo Proteine (C.I.R.M.M.P.), University of Florence, Via L. Sacconi 6, 50019 Sesto Fiorentino, Italy

**Keywords:** High potential iron–sulfur proteins, Metalloproteins, Paramagnetic NMR, Fast nuclear relaxation

## Abstract

High potential iron–sulfur proteins (HiPIPs) are a class of small proteins (50–100 aa residues), containing a 4Fe–4S iron–sulfur cluster. The 4Fe–4S cluster shuttles between the oxidation states [Fe_4_S_4_]^3+/2+^, with a positive redox potential in the range (500–50 mV) throughout the different known HiPIPs. Both oxidation states are paramagnetic at room temperature. HiPIPs are electron transfer proteins, isolated from photosynthetic bacteria and usually provide electrons to the photosynthetic reaction-center. PioC, the HIPIP isolated from *Rhodopseudomonas palustris* TIE-1**,** is the smallest among all known HiPIPs. Despite their small dimensions, an extensive NMR assignment is only available for two of them, because paramagnetism prevents the straightforward assignment of all resonances. We report here the complete NMR assignment of ^1^H, ^13^C and ^15^N signals for the reduced [Fe_4_S_4_]^2+^ state of the protein. A set of double and triple resonance experiments performed with standardized parameters/datasets provided the assignment of about 72% of the residues. The almost complete resonance assignment (99.5% of backbone and ca. 90% of side chain resonances) was achieved by combining the above information with those obtained using a second set of NMR experiments, in which acquisition and processing parameters, as well as pulse sequences design, were optimized to account for the peculiar features of this paramagnetic protein.

## Biological context

High potential iron proteins (HiPIPs), together with ferredoxins are a group of metalloproteins that contain a 4Fe–4S cubane cluster coordinated by four cysteine residues. HiPIPs are electron transfer proteins with positive reduction potentials in the range + 50/ + 500 mV vs SHE (Heering et al. 1995). In bacteria HiPIPs are assembled in the cytoplasm and then translocated as holoproteins to the periplasm via the TAT translocation system (Bruser et al. 2003). HiPIPs are mainly found in purple photosynthetic bacteria where they mediate electron transfer between cytochrome bc1 and the photosynthetic reaction center (Van Driessche et al. 2003).

*Rhodopseudomonas palustris* displays one of the highest metabolic versatilities known. It can perform any of the four modes of metabolism: photoautotrophic, photoheterotrophic, chemoautotrophic and chemoheterotrophic (Larimer et al. 2004). The strain *R. palustris* TIE-1 is 99% identical to the type strain and displays the capacity of growing by photoferrotrophism obtaining electrons for its photosynthetic metabolism from extracellular iron-minerals instead of water as chloroplasts do (Jiao et al. 2005). This is proposed to be one of the most ancient forms of bioenergetic metabolism, giving rise to the banded-iron formations (BIFs) with high abundance of ferric iron in the late Archaean oceans (Widdel et al. 1993). The photoferrotrophic metabolism of *R. palustris* TIE-1 is mediated by the Pio (phototrophic iron oxidation) operon that contains three proteins PioA, PioB and PioC (Jiao and Newman 2007). PioC is proposed to mediate the connection between the iron oxidase at the surface of the bacteria (PioA) and the reaction center in the inner membrane (Bird et al. 2011). Its reduction potential is + 450 mV vs SHE and it is proposed to participate in non-cyclic photosynthesis (Bird et al. 2014). In order to investigate the network of interactions that underpin the biological activity of PioC we undertook the assignment of the NMR signals in this protein.

## Methods and experiments

PioC was expressed and purified as previously reported (Bird et al. 2014). Samples of PioC were produced as unlabeled, single ^15^N-labeled, double ^13^C and ^15^N-labeled samples. In all cases, the expression and purification protocol were identical throughout except in the addition of ammonium sulfate (^15^N_2_, 99%) (1 g/l) and [U–^13^C_6_] d-glucose (2 g/l) in the M9 minimal media when labeling was required. BL21 DE3 cells were double transformed with pET32h, a plasmid containing the construct thioredoxin-6xHis-thrombin cleavage site-PioC, and with pDB1281, a plasmid that carries the machinery for the assembly of iron–sulfur clusters. Cells were grown in Luria–Bertani (LB) supplemented with 100 mg/dm^3^ ampicillin and 35 mg/dm^3^ chloramphenicol until the OD_600 nm_ of 0.6, then they were induced with 1.0 mM arabinose, 20 μM FeCl_3_ and 200 μM cysteine were also added. Cells were again incubated until the OD_600 nm_ of 1 and then harvested and washed in M9 minimal media salts before resuspension in M9 minimal media. Once re-suspended, cells were incubated for one hour before induction with 0.5 mM IPTG. After 4 h cells were harvested by centrifugation and disrupted using a French Press at 1000 psi. The lysate was ultra-centrifuged at 204,709 × g for 90 min at 4 °C to remove cell membranes and debris and the supernatant was dialyzed overnight against 50 mM potassium phosphate buffer pH 5.8 with 300 mM NaCl before injection in a His-trap affinity column (GE Healthcare). The fraction containing Histag-PioC eluted with 250 mM imidazole and was incubated overnight with Thrombin (GE Healthcare) for digestion. The final purified PioC (His-tag free) was then concentrated from the flow through of a second passage through the His-trap column using an Amicon Ultra Centrifugal Filter (Millipore) with a 3 kDa cutoff. The purity of PioC was confirmed by SDS-PAGE with Blue Safe staining (NzyTech) and by UV–Visible spectroscopy.

All experiments were recorded using Bruker AVANCE-NEO spectrometers operating at 700 MHz and 500 MHz, equipped with cryogenically cooled triple resonance inverse detection probe heads (CP-TXI), except ^13^C-detected experiments, which were acquired at 176.05 MHz using a cryogenically cooled probe-head optimized for ^13^C direct detection (CP-TXO), and ^1^H experiments which were recorded at 400 MHz using a room temperature, selective 5 mm ^1^H probe without pulsed field gradients. All spectra were processed using the Bruker software TopSpin. For the assignment of diamagnetic resonances, a set of double and triple resonance experiments was performed: ^13^C- and ^15^N-HSQC and HSQC-NOESY, HNCA, HNCO, HNCACO, CBCACONH, CBCANH, HBHANH, HCCH-TOCSY (Cavanagh et al. 2007). For this set of experiments, radio frequency pulses, carrier frequencies, acquisition and processing parameters were taken as normally done in biomolecular NMR studies. Furthermore, to identify signals affected by paramagnetic relaxation, we performed double and triple resonance experiments using parameters optimized to identify signals with high R_1_ and R_2_ values (Arnesano et al. 2006). The ^15^N HSQC spectrum was acquired using INEPT and recycle delays as short as 800 us and 150 ms, respectively  (Banci et al. 2013). Acquisition times were shortened to 47 ms (t_2_) and 27 ms (t_1_), the inverse INEPT step following ^15^N evolution was removed and signal acquired in antiphase mode without ^1^H decoupling during t_2_ acquisition (Ciofi-Baffoni et al. 2014). Spectral windows were enlarged: respectively 80 ppm and 22 ppm were used in ^15^N and ^1^H dimensions. On average, 512 scans/fid were acquired. Also, the HNCA was tailored to identify Cα connectivities of cluster-bound cysteines, whose Cα and Cβ resonances are expected to be shifted, due to hyperfine contact shift, in the 110–70 ppm region (Bertini et al. 1994a). Proton resonances were calibrated with respect to the signal of 2,2-dimethylsilapentane-5-sulfonic acid (DSS). Nitrogen chemical shifts were referenced indirectly to the ^1^H standard using a conversion factor derived from the ratio of NMR frequencies. Carbon resonances were calibrated using the signal of dioxane at 69.4 ppm (298 K) as secondary reference. Assignments were performed with the program CARA (https://www.nmr.ch). Backbone dihedral angles and secondary structure propensities were predicted with the program TALOS-+ (Shen et al. 2009).

## Assignments and data deposition

**Fig. 1:**
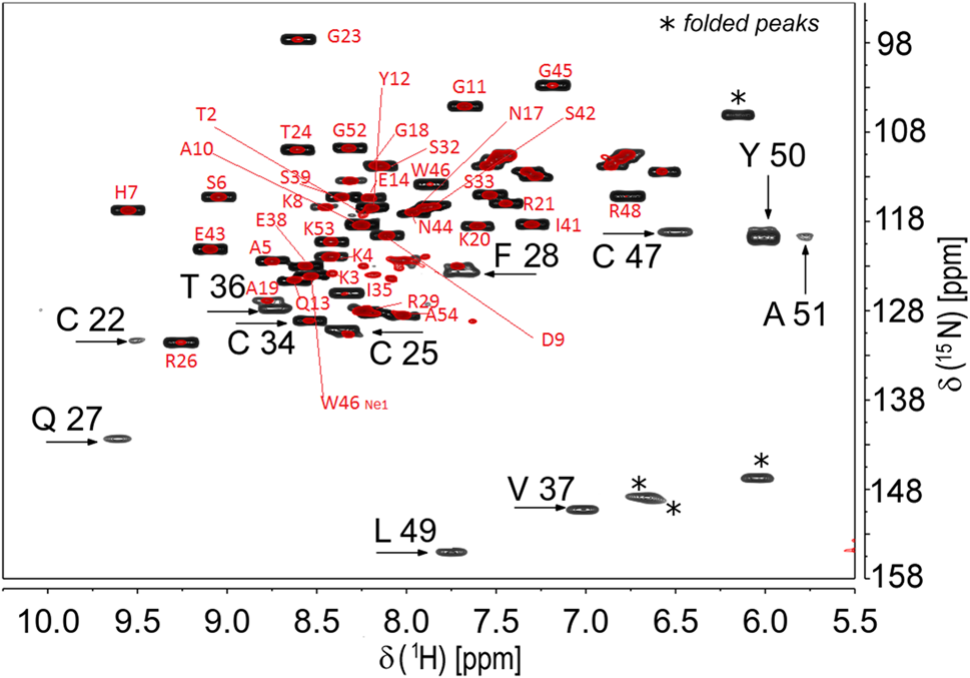
Red colored: 2D ^1^H–^15^N HSQC spectrum of ^15^N-labeled PioC collected at 298 K on a Bruker Avance NEO 500 MHz spectrometer. Backbone resonance assignments are labeled with single letter amino acid code followed by their sequence numbers. Black colored: 2D ^1^H–^15^N HSQC-AP spectrum, collected as described in text. Signals that are visible only in the tailored ^1^H–^15^N HSQC-AP experiments are indicated with arrow and labeled in black, using larger characters

A complete set of NMR experiments performed using standard parameters used for biomolecular NMR, described in the previous section, led to the identification and to the sequential assignment of protein fragments: Thr2-Arg21, Arg29-Cys34, Glu38-Trp46 and Gly52-Ala54, corresponding to only 72% of residues (38 out of 53 residues, excluding Ala1). The unassigned fragments: Cys22-Phe28, Ile35-Val37 and Cys47-Ala51, correspond to the four cysteine residues binding the 4Fe–4S cluster (Cys22, Cys25, Cys34 and Cys47) and the three amino acids following each of the four cluster-binding Cys residues. The symmetry breaking exceptions are the cases of Cys34, which can be identified thanks to its link with the preceding amino acid, and Ala51, which is completely undetectable in standard double and triple resonance experiments. The effects of paramagnetism are also clearly reflected in the ^15^N HSQC spectrum shown in Fig. 1, reporting the assignments performed with this set of experiments, in which only 39 backbone peaks were observed out of 49 non-proline residues.

In order to recover signals that escaped detection due to fast relaxation, to fill the gaps in the sequence specific assignment and to identify all protein resonances, a second set of NMR experiments was performed with parameters optimized to identify fast relaxing resonances, as well as signals falling in “unexpected” spectral regions. As a sample case, we report in Fig. 1 the comparison of the ^15^N-HSQC recorded with standard parameters with a “paramagnetism-tailored” ^15^N-HSQC. The optimization of the ^15^N-HSQC relies on a number of adjustments (Ciofi-Baffoni et al. 2014): the decrease of the INEPT transfer from 2.75 ms to values below 1 ms (typically 600–800 μs); the removal the inverse INEPT transfer delay and the signal acquisition in the antiphase mode; the substitution of water selective pulses during the Watergate with the binomial 3–9–19 ^1^H 180° pulse (Mori et al., 1995); the use of states or states-TPPI instead of the echo–antiecho quadrature detection; the use of pulsed field gradients as short as 200 μs; very short recycle delay in order to suppress water signal via progressive saturation and to collect a very large number of scans within reasonable experimental time; adjust the number of t_1_ and t_2_ points according to the relaxation properties of “target” resonances. Thanks to these adjustments, the 10 HN resonances that were missing in the standard experiments could be identified. This procedure was applied also on HNCA, HNCO, CBCACONH (Gelis et al. 2003), thus allowing us to detect sequential connectivities from all Cys Cα atoms and from the fast relaxing HN residues observed with the paramagnetism-optimized ^15^N HSQC. A few one dimensional ^1^H NOE experiments and a set of ^13^C direct detected experiments CACO, CON, CC-COSY, contributed to fill the gaps in the assignment (Balayssac et al. 2006). Overall, we obtained (excluding the N-ter Val1) 100% of backbone ^1^HN, ^13^C, and ^15^N resonances, 98% of Hα (Cys47 Hα is missing), 86% and 91% of ^1^H and ^13^C side chains atoms, respectively. The complete assignment is deposited in BMRB, ID 34487.

**Fig. 2:**
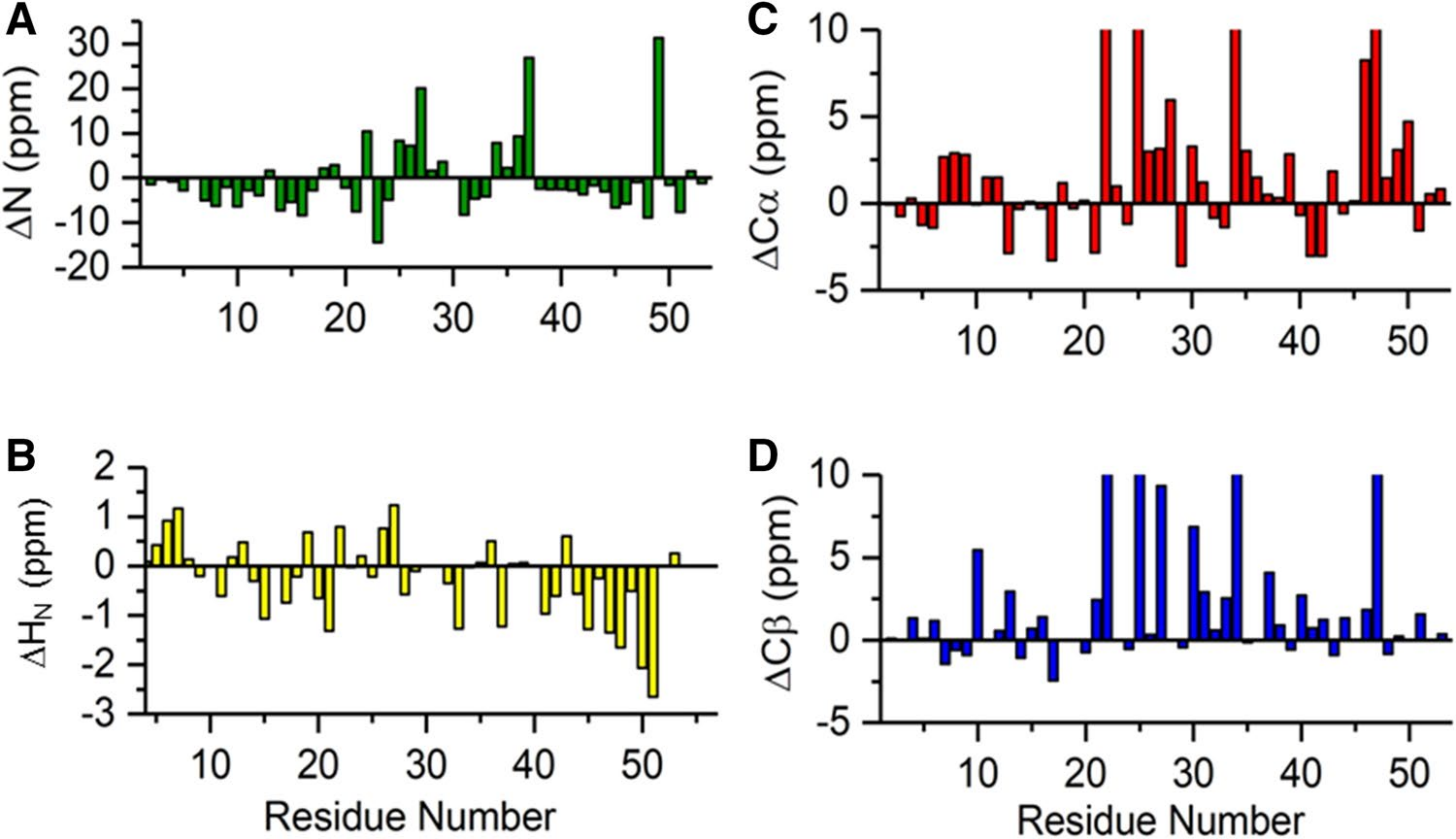
Chemical shift differences between values observed in PioC (298 K) and random coil values. **a** Backbone nitrogen atoms, **b** backbone amide H_N_ protons, **c** backbone ^13^Cα, and **d** side chain ^13^Cβ

Paramagnetism does not affect only the NMR assignment, but also the analysis of chemical shifts. On the one hand, the use of chemical shifts to obtain information on secondary structure elements must be “handled with care”, because contact and pseudocontact contributions may affect chemical shifts much more than secondary structure indexes. On the other hand, the deviations from random coil values directly point out important aspects of the system under investigation and correlate with structural properties of the environment of the cluster. Figure 2 shows the plot of chemical shift differences from random coil values, obtained with the program ncSPC (neighbor corrected structural propensity calculator) (Tamiola et al. 2010). They are usually termed as “secondary chemical shifts”, however in this case this definition is largely misleading. The plot of nitrogen ΔN shifts (Fig. 2a) shows three values whose deviation from expectation are larger than 15 ppm and therefore they can only be interpreted as evidence of the formation of three hydrogen bonds between amide groups and electron donor sulfur atoms (either the Sγ of cysteine residues bound to the cluster or an inorganic sulfide ion of the cluster) (Lin et al. 2005). The solution structure of PioC shows indeed that three hydrogen bonds link the HN groups of Gln27, Val37 and Leu49 with the Sγ of the preceding cluster bound cysteine, respectively Cys25, Cys34 and Cys47 (Trindade et al. submitted). Also ΔH_N_ shifts have a peculiar behavior, because it appears from Fig. 2b that the last 10 aminoacids show a consistent trend of upfield shifted values, a trend that is also confirmed by ΔN values. This feature, which has been indeed previously observed also for two other HiPIPs from different bacterial sources (Bertini et al. 1994b; Banci et al. 1995), could be due to ring current effects provided by the two aromatic residues W46 and Y50. Albeit the relatively low sequence homology among the different HiPIPs (Van Driessche et al. 2003), the presence of a Trp and of a Tyr/Trp/Phe residues in the position Cys_4_(i − 1) and Cys_4_(i + 2 or i + 3) is a well conserved features among HiPIPs. These two aromatic residues are supposed to play a key role in providing a hydrophobic environment that can stabilize the 4F–-4S cluster and give rise to the very high redox potential of the [Fe_4_S_4_]^3+/2+^ redox pair. This shielding effect gives rise to the upfield shifts experienced by the HN vectors of the C-term part of the protein.

The electron delocalization via σ bond from the 4Fe–4S cluster to the bound cysteines originates the strong deviation from the random coil shifts observed for both Cβ and Cα (Fig. 2c, d). Indeed, ΔCβ and ΔCα of Cys residues are fully due to contact hyperfine contributions. Pseudocontact contribution are expected to be negligible because the electronic distribution and the symmetry of the cluster is such that we do not expect a significant magnetic anisotropy in the system (Bertini et al. 1992). Indeed, once the few strong contact contributions are accounted for, then the observed chemical shift differences can be considered as “true” secondary shifts. Their analysis shows that only a small α-helix in the N-term region is observed in residues 7–11.

## Conclusion

The complete NMR assignment of the HiPIP PioC from *R. palustris* TIE-1 is a mandatory step in order to obtain the solution structure of the protein. However, we have shown here how some key features of HiPIP proteins can be observed, discussed and interpreted also on the basis of chemical shifts. Hydrogen bonds from the NH backbone groups of Cys(i + 2) or Cys(i + 3) residues are important to define the consensus sequence around the 4Fe–4S cluster. Furthermore, the aromatic residues surrounding the last cluster-bound Cys residue (in the present case Cys47), in the C-term part of the protein, provide a shielding environment that is probably crucial to determine the unique properties of HiPIPs. The protein has no clear secondary structure elements, suggesting that its folding properties are driven by the cluster. Intriguingly, PioC is a very nice example of how the assignment of a small polypeptide chain (54 aa) may turn into a real puzzle as soon as the polypeptide is bound to a paramagnetic metal or, like in the present case, to a polymetallic cluster. The present assignment is a useful toolkit to study the NMR properties of other small metalloproteins or de novo designed metalloproteins (Kim et al. 2018).

## Data Availability

Assignment deposited in BMRB, ID 34487.
